# Proton-pump inhibitor use amongst patients with severe hypomagnesemia

**DOI:** 10.3389/fphar.2023.1092476

**Published:** 2023-01-30

**Authors:** Sherry Seah, Yen Kheng Tan, Kevin Teh, Wann Jia Loh, Pei Ting Tan, Leng Chuan Goh, Roy Debajyoti Malakar, Tar Choon Aw, Chin Shern Lau, Trishpal Dhalliwal, Swee Leng Kui, Jia Wen Kam, Joan Khoo, Tunn Lin Tay, Eberta Tan, Vanessa Au, Shui Boon Soh, Meifen Zhang, Thomas F. King, Linsey Gani, Troy H. Puar

**Affiliations:** ^1^ Yong Loo Lin School of Medicine, National University of Singapore, Singapore, Singapore; ^2^ Doctor of Medicine Programme, Duke-NUS (National University School) Medical School, Singapore, Singapore; ^3^ Department of Gastroenterology, Changi General Hospital, Singapore, Singapore; ^4^ Department of Endocrinology, Changi General Hospital, Singapore, Singapore; ^5^ Department of Clinical Trial Research Unit, Changi General Hospital, Singapore, Singapore; ^6^ Department of Pharmacy, Changi General Hospital, Singapore, Singapore; ^7^ Department of Renal Medicine Changi General Hospital, Singapore, Singapore; ^8^ Department of Laboratory Medicine, Changi General Hospital, Singapore, Singapore; ^9^ Department of Internal Medicine, Changi General Hospital, Singapore, Singapore; ^10^ Department of Cardiology, Changi General Hospital, Singapore, Singapore

**Keywords:** omeprazole, magnesium, chronic kidney disease, drug adverse effects, medication safety, toxicity

## Abstract

**Introduction:** Long-term proton pump inhibitor (PPI) use has been associated with hypomagnesemia. It is unknown how frequently PPI use is implicated in patients with severe hypomagnesemia, and its clinical course or risk factors.

**Methods:** All patients with severe hypomagnesemia from 2013 to 2016 in a tertiary center were assessed for likelihood of PPI-related hypomagnesemia using Naranjo algorithm, and we described the clinical course. The clinical characteristics of each case of PPI-related severe hypomagnesemia was compared with three controls on long-term PPI without hypomagnesemia, to assess for risk factors of developing severe hypomagnesemia.

**Results:** Amongst 53,149 patients with serum magnesium measurements, 360 patients had severe hypomagnesemia (<0.4 mmol/L). 189 of 360 (52.5%) patients had at least possible PPI-related hypomagnesemia (128 possible, 59 probable, two definite). 49 of 189 (24.7%) patients had no other etiology for hypomagnesemia. PPI was stopped in 43 (22.8%) patients. Seventy (37.0%) patients had no indication for long-term PPI use. Hypomagnesemia resolved in most patients after supplementation, but recurrence was higher in patients who continued PPI, 69.7% *versus* 35.7%, *p* = 0.009. On multivariate analysis, risk factors for hypomagnesemia were female gender (OR 1.73; 95% CI: 1.17–2.57), diabetes mellitus (OR, 4.62; 95% CI: 3.05–7.00), low BMI (OR, 0.90; 95% CI: 0.86–0.94), high-dose PPI (OR, 1.96; 95% CI: 1.29–2.98), renal impairment (OR, 3.85; 95% CI: 2.58–5.75), and diuretic use (OR, 1.68; 95% CI: 1.09–2.61).

**Conclusion:** In patients with severe hypomagnesemia, clinicians should consider the possibility of PPI-related hypomagnesemia and re-examine the indication for continued PPI use, or consider a lower dose.

## Introduction

Proton-pump inhibitors (PPI) are one of the most widely prescribed medications. While PPI are generally well-tolerated, they have been linked to increased risk of fractures, dementia, infections, and hypomagnesemia (Sheen and Triadafilopoulos, 2011). Since Epstein first described a case of severe hypomagnesemia with PPI use in 2006 (Epstein et al., 2006), other cases or case series have been reported (Hess et al., 2012; Danziger et al., 2013; Janett et al., 2015; Pasina et al., 2016). While some case-control and cross-sectional studies have demonstrated an association of PPI use with hypomagnesemia, other studies have not (Faulhaber et al., 2013; Koulouridis et al., 2013; Markovits et al., 2014; Van Ende et al., 2014; Zipursky et al., 2014; Kim et al., 2015; Pisani et al., 2016; Biyik et al., 2017). These conflicting findings may lead to uncertainty amongst clinicians of this potential adverse effect of PPI. Severe hypomagnesemia can lead to life-threatening arrythmias or seizures, and it is important to prevent recurrent episodes. Previous studies did not conduct a detailed review of individual patients to assess for causality, and it is currently unknown how frequent severe hypomagnesemia could be attributed to PPI use, or if appropriate management is taken to avoid recurrence of hypomagnesemia.

PPI-related hypomagnesemia appears to occur only after prolonged use of PPI, usually at least 3 months (Pisani et al., 2016). Current understanding is that PPI may impair gastrointestinal absorption of magnesium, *via* alteration of intestinal mucosal pH and interference with transient potential melastatin-6 (TRPM6)-mediated active absorption of magnesium (Bai et al., 2012; Lameris et al., 2015). In a prospective study by Begley et al. (2016), patients using a prolonged course of PPI were monitored prospectively for 8 months, with none of the patients developing clinically significant hypomagnesemia. This suggests that hypomagnesemia may only affect a small proportion of patients with genetic or clinical predisposition (Pisani et al., 2016). Previously identified risk factors include chronic renal impairment or concomitant use of diuretics (Danziger et al., 2013).

Amongst patients with severe hypomagnesemia, we aimed to assess the likelihood of PPI-related hypomagnesemia using the Naranjo algorithm (Naranjo et al., 1981), and described the clinical course of patients with at least possible PPI-related severe hypomagnesemia. We subsequently aimed to identify risk factors for developing severe hypomagnesemia amongst long-term PPI users, by comparing these cases with matched controls of patients on long-term PPI without hypomagnesemia.

## Materials and methods

We conducted a retrospective study of all patients treated at a single tertiary center, Changi General Hospital, Singapore, from 2013 to 2016. Local ethics approval was obtained, and waiver of consent was granted for all patients. The study was approved by the Singapore Health Services ethics committee and institutional review board (IRB 2017/2112), and is registered with Clinicaltrials.gov (NCT04426994).

### Patients with severe hypomagnesemia assessed for PPI-related hypomagnesemia

**FIGURE 1:**
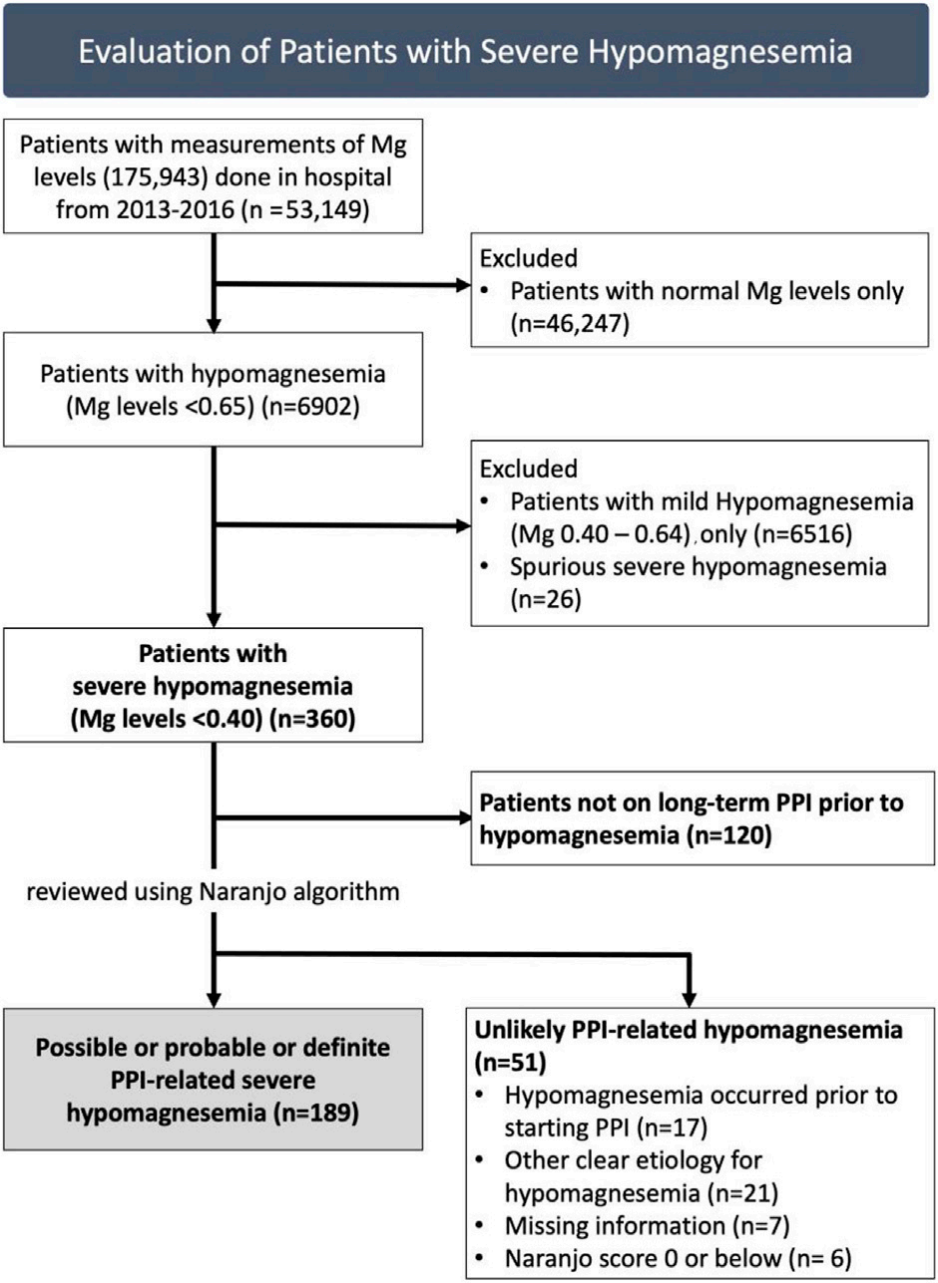
Patients with severe hypomagnesemia assessed for likelihood of proton-pump inhibitor (PPI)-related hypomagnesemia, with 189 identified to have at least possible PPI-related severe hypomagnesemia.

We identified all patients with documented severe hypomagnesemia from 2013 to 2016. 175,943 measurements of serum magnesium were taken in 53,149 patients during this period. 6,902 patients had at least one episode of hypomagnesemia (<0.65 mmol/L). The first episode of severe hypomagnesemia (<0.4 mmol/L) was taken for patients with more than one episode. All patients were admitted during the episode of severe hypomagnesemia, and reconciliation of medication use was conducted by a pharmacist upon admission. For evaluation of PPI-related hypomagnesemia, we included patients on long-term PPI use (at least 3 months) prior to development of severe hypomagnesemia. We excluded patients with spurious hypomagnesemia results (Figure 1).

All patients were evaluated using the Naranjo algorithm (Naranjo et al., 1981) which indicates likelihood of an adverse drug reaction due to PPI: ≤0 doubtful, one to four possible, five to eight probable, and ≥9 definite (details in Supplementary Material). All patients were scored by two investigators (S.S and T.P), and discrepancies were settled by mutual consensus, or by a third investigator (Y.K.T). Electronic medical records were comprehensive, including data of previous medication prescriptions, laboratory tests from year 2000 onwards, from both tertiary and primary healthcare systems. Patients were assessed for any recurrence or resolution of hypomagnesemia until 30^th^ September 2017. Medical information obtained included demographics, Charlson’s Comorbidity Index (CCI) (Charlson et al., 1987), concomitant medications, and laboratory data (including serum calcium, serum magnesium, urine magnesium, serum potassium, parathyroid hormone, where available). Glomerular filtration rate was estimated (eGFR) using CKD-EPI equation (Levey et al., 2009). In patients with severe hypomagnesemia, any associated complications were noted, including development of hypokalemia, hypocalcemia, arrthythmias and seizures.

Information was collected regarding magnesium levels prior to PPI initiation, and action (if any) taken after hypomagnesemia occurred (withdrawal, or continuation of PPI). We used the NICE guidelines to define standard-dosage PPI (e.g. omeprazole daily dose of 20 mg or equivalent) (NICE, 2014), and dosages above that as high-dosage PPI. We assessed if patients had an indication for long-term PPI use (Freedberg et al., 2017). This was defined as single antiplatelet therapy (SAPT) with additional risk factors including (Sheen and Triadafilopoulos, 2011) dual antiplatelet therapy (DAPT), Epstein et al. (2006) concomitant anticoagulant, NSAIDs or steroid use, Hoorn et al. (2010) history of gastrointestinal bleeding or ulcer, or (Pasina et al., 2016) age above 65 years; or gastroesophageal reflux disease (GERD), complicated by either Barrett’s esophagus or esophagitis (Freedberg et al., 2017).

### Risk factors for developing hypomagnesemia

To identify risk factors for developing severe hypomagnesemia, we compared the patients identified with at least possible PPI-related severe hypomagnesemia (cases), with patients on long-term PPI without hypomagnesemia (controls). Controls were patients prescribed long-term PPI at the same center over the same period, without documented hypomagnesemia. A total of 649,374 prescriptions of PPI were given to 158,523 patients from 2013 to 2016. We excluded prescriptions given for a duration of fewer than 3 months, pro re nata (when necessary), and patients with hypomagnesemia. We randomly selected three controls per case, matched for the date of PPI prescription. Duration of PPI therapy was date of first-ever PPI prescription till most recent prescription, or date of hypomagnesemia (if occurred).

### Statistical analysis

Statistical analyses were performed using SPSS version 25, IBM Corp., Armonk, New York, United States.

Continuous variables were expressed as mean (SD), or median (IQR) and compared using independent *t*-test, or Mann-Whitney test as appropriate. Categorical variables were compared using Chi-square test. Spearman correlation was used to compare the association between serum magnesium, with calcium and potassium, during the episode of severe hypomagnesemia. We conducted backwards stepwise selection process. For risk factors of severe hypomagnesemia, variables associated with severe hypomagnesemia in the univariate analysis (*p* < 0.1) or previously described in the literature were included in the multivariate logistic regression model. Missing values were imputed for the purpose of the multivariate logistic regression model, with median values used. Individual components of CCI were used in the multivariate model. Statistical significance was set at *p* less than 0.05.

## Results

Of 175,943 magnesium measurements done in 53,149 patients from 2013 to 2016, there were 360 patients with severe hypomagnesemia (Figure 1). We excluded 120 patients who were not taking PPI prior to hypomagnesemia. Hence, we reviewed 240 patients with severe hypomagnesemia for PPI-related hypomagnesemia. Fifty-one patients were deemed unlikely to have PPI-related hypomagnesemia: 17 patients with hypomagnesemia which occurred prior to starting PPI, 21 patients with other clear etiology for hypomagnesemia (e.g. gastrointestinal malignancy, chronic alcoholism), 7 patients with missing information and six patients with Naranjo score of 0 or below (doubtful for PPI-related hypomagnesemia).

In total, 189 of 360 (52.5%) patients with severe hypomagnesmia were identified to have at least possible PPI-related hypomagnesemia using Naranjo algorithm: 128 possible, 59 probable, two definite. The mean age was 71.8 [11.0] years, with 103 females (54.5%) (Table 1). Majority of patients were using omeprazole (97.9%), with a median duration of PPI use of 57 (28–86) months. At baseline, between patients with lower (<5) or higher (≥ 5) Naranjo scores, there were no differences in demographics, co-morbidities, duration or dose of PPI.

**TABLE 1:** Baseline Characteristics of 189 patients using proton pump inhibitor (PPI) prior to severe hypomagnesemia, evaluated with Naranjo algorithm for likelihood of PPI-related hypomagnesemia, 1–4 (possible), 5–8 (probable), ≥9 (definite).

	Possible (*n* = 128)	Probable or definite (n = 61)	Total (n = 189)	*p*
Age	71.3 ± 10.9	72.9 ± 11.2	71.8 ± 11.0	0.38
Female	71 (55.5%)	32 (52.5%)	103 (54.5%)	0.70
Race				0.80
Chinese	75 (58.6%)	38 (62.3%)	113 (59.8%)	
Malay	34 (26.6%)	17 (27.9%)	51 (27.0%)	
Indian	11 (8.6%)	3 (4.9%)	14 (7.4%)	
Others	8 (6.3%)	3 (4.9%)	11 (5.8%)	
BMI, n = 142	23.3 ± 5.6	23.6 ± 4.8	23.3 ± 5.3	0.78
eGFR, n = 180	43.6 ± 37.8	71.3 ± 38.2	52.2 ± 40.0	<0.001
Haemoglobin, n = 177	10.9 ± 2.2	11.0 ± 2.0	11.0 ± 2.2	0.81
Diabetes	99 (77.3%)	43 (70.5%)	142 (75.1%)	0.31
Hypertension	109 (85.2%)	56 (91.8%)	165 (87.3%)	0.20
Hyperlipidemia	90 (70.3%)	47 (77.0%)	137 (72.5%)	0.33
Charlson’s Comorbidity Index	6 (3–9)	6 (4–8)	6 (3–9)	0.73
Ischemic Heart Disease	85 (66.4%)	40 (65.6%)	125 (66.1%)	0.91
Atrial Fibrillation	13 (10.2%)	12 (19.7%)	25 (13.2%)	0.071
Type of PPI (omeprazole)	126 (98.4%)	59 (96.7%)	185 (97.9%)	0.44
High Dose PPI	99 (77.3%)	42 (68.9%)	141 (74.6%)	0.21
Duration of PPI (months), n = 188	59 (32–95)	52 (22–79)	57 (28–86)	0.16

BMI, body mass index; eGFR, estimated glomerular filtration rate; PPI, proton pump inhibitor.

Data presented as mean (SD), median (interquartile range), or number (percentage).

*p:* comparison between possible *versus* probable/definite.

Patients with at least probable PPI-related hypomagnesemia (Naranjo score ≥ 5) were more likely to have no alternative etiology for hypomagnesemia, compared to those with possible PPI-related hypomagnesemia, 75.4% *versus* 3.1%, *p* < 0.001 (Table 2). Overall, 39.7% of patients were using diuretic therapy, 66.7% had renal impairment, and 20.6% had a history of gastrointestinal loss prior to development of hypomagnesemia. PPI therapy was stopped in only 43 of 189 patients (22.8%) of patients after the episode of severe hypomagnesemia, and this was more often in patients with probable PPI-related hypomagnesemia, 47.5%, compared with possible PPI-related hypomagnesemia, 10.9%, *p* < 0.001. Forty-two patients were switched to a histamine-2 receptor antagonist, while one patient had cessation of PPI therapy. Only 63% of cases had a strong indication for long-term PPI use, which was most often SAPT usage in a patient above 65 years.

**TABLE 2:** Etiology and Outcomes of 189 patients with proton pump inhibitor (PPI)-related severe hypomagnesemia as evaluated with Naranjo algorithm, 1–4 (possible), 5–8 (probable), ≥9 (definite).

	Possible (n = 128)	Probable or definite (n = 61)	Total (n = 189)	*p*
No other possible etiology for Hypomagnesemia	4 (3.1%)	46 (75.4%)	50 (26.5%)	<0.001
Diuretic Use	63 (49.2%)	12 (19.7%)	75 (39.7%)	<0.001
Renal impairment (eGFR <60 mL/min)	98 (79.0%)	22 (39.3%)	120 (66.7%)	<0.001
Gastrointestinal losses	33 (25.8%)	6 (9.8%)	39 (20.6%)	0.011
PPI stopped	14 (10.9%)	29 (47.5%)	43 (22.8%)	<0.001
PPI re-challenge	1/1 (100%)	6/6 (100%)	7/7 (100%)	-
Previous milder hypomagnesemia	97 (88.2%)	58 (96.7%)	155 (91.2%)	0.062
Indication for PPI usage	76 (59.4%)	42 (70.5%)	119 (63.0%)	0.14
Indication for PPI was use of SAPT and one of the following				
- DAPT	22 (17.2%)	15 (24.6%)	37 (19.6%)	0.23
- Anticoagulant or NSAIDs	7 (5.5%)	6 (9.8%)	13 (6.9%)	0.27
- History of BGIT or gastric ulcer	9 (7.0%)	5 (8.2%)	14 (7.4%)	0.78
- >65 years old	63 (49.2%)	38 (62.3%)	101 (53.4%)	0.092

eGFR, estimated glomerular filtration rate; PPI, proton pump inhibitor; SAPT, single anti-platelet therapy; DAPT, dual anti-platelet therapy; NSAIDs, non-steroidal anti-inflammatory drugs; BGIT, bleeding from gastro-intestinal tract.

All patients were given oral or intravenous magnesium replacement during the admission, and most patients had resolution of hypomagnesemia within a few days. Patients who stopped using PPI were less likely to develop a subsequent episode of hypomagnesemia, compared to those who were continued on PPI therapy, five of 14 (35.7%), *versus* 115 of 165 (69.7%), *p* = 0.009. Out of the five patients who stopped PPI but had recurrent hypomagnesemia, three patients had alternative etiologies (renal impairment, diuretic use) to account for this.

Overall, 56.4% of patients developed hypocalcaemia, while 40.2% developed hypokalaemia (Table 3). In addition, this was complicated by an episode of arrhythmia in 4.8% of patients, and seizure in 5.3%. There was a weak positive correlation between magnesium and calcium, *r*_s_ = 0.360, *p* < 0.001, while there was no correlation between magnesium and potassium, *r*_s_ = −0.008, *p* = 0.109 (Supplementary Figure S1A and S1B). In 13 patients, urine Mg was measured, which were normal/low in all but two patients, one of whom was on a diuretic and had renal impairment. In 32 patients with hypocalcaemia, iPTH was normal in 10 patients, and elevated in 22 patients, of whom 86.3% had renal impairment.

**TABLE 3:** Characteristics during severe Hypomagnesemia amongst 189 patients with proton pump inhibitor (PPI)-related hypomagnesemia.

Biochemical test	Result
Urine mg, mmol/L (n = 13)	2.5 ± 2.6
Urine Mg, References range 1.97–4.94 mmol/day	
- Low	7 (53.8%)
- Normal	4 (30.8%)
- High	2 (15.4%)
iPTH, mmol/L (n = 41)	16.8 ± 17.4
Hypocalcemia patients with iPTH results (n = 33)	
- iPTH low	0 (0%)
- iPTH normal	10 (30.3%)
- iPTH high	23 (69.7%)
Normocalcemia patients with iPTH results (n = 7)	
- iPTH low	1 (14.3%)
- iPTH normal	2 (28.6%)
- iPTH high	4 (57.1%)
Hypocalcemia	92 (56.4%)
Hypokalemia	70 (40.2%)
Episode of arrythmia	9 (4.8%)
Episode of seizure	10 (5.3%)
Days required to recover to normal Mg (n = 142)	1.0 (0–2) and 1.0

Mg, magnesium; iPTH, parathyroid hormone.

**FIGURE 2:**
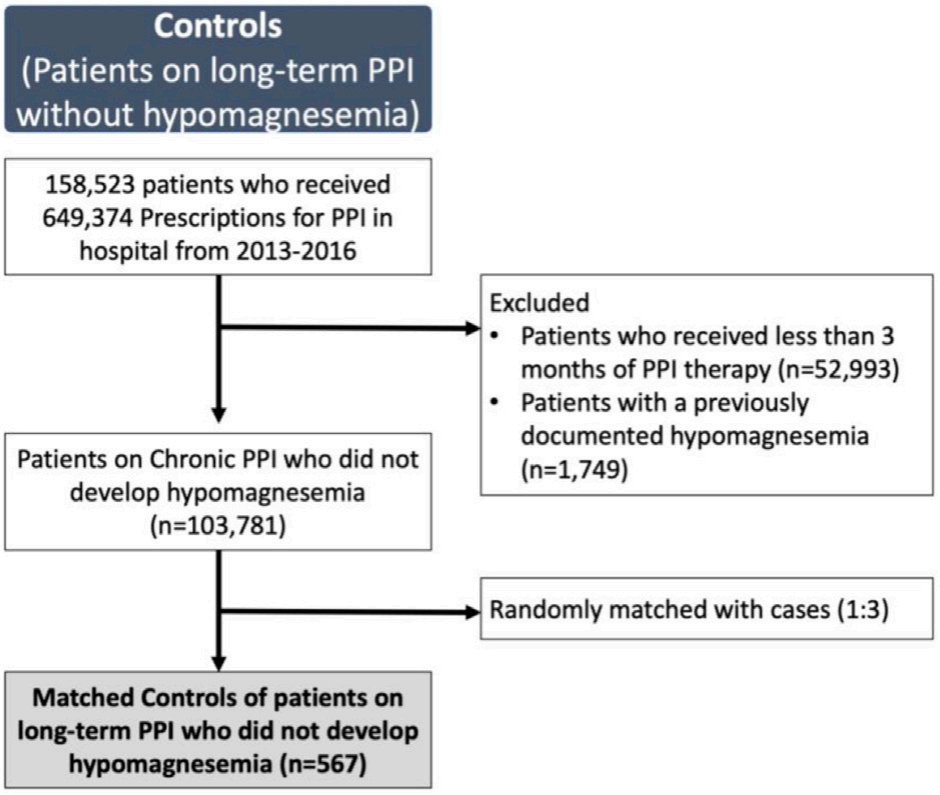
Patients on long-term proton-pump inhibitor (PPI) without documented hypomagnesemia selected as controls (*n* = 567)

Each of the 189 cases with severe hypomagnesemia were matched with three controls, who were on PPI therapy without documented hypomagnesemia (Figure 2). On univariate analysis, patients with severe hypomagnesemia were more likely to be older (OR, 1.04; 95% CI, 1.02–1.05), female (OR, 1.85; 95% CI, 1.35–2.61), lower BMI (OR, 0.95; 95% CI 0.92–0.99), on high dose PPI (OR, 2.10; 95% CI, 1.46–3.04), renal impairment (OR, 4.21; 95% CI, 2.88–6.16), and had a higher CCI score (OR, 1.77; 95% CI, 1.61–1.96) (Table 4). On multivariate analysis, variables included were age, gender, ethnicity, BMI, PPI dosage, duration of PPI, renal impairment, use of diuretics, CCI score and presence of diabetes. The significant risk factors on multivariate analysis for severe hypomagnesemia were female gender (OR 1.73; 95% CI, 1.17–2.57), presence of diabetes mellitus (OR, 4.62; 95% CI, 3.05–7.00), low BMI (OR, 0.90; 95% CI, 0.86–0.94), high dose PPI therapy (OR, 1.96; 95% CI, 1.29–2.98), renal impairment (OR, 3.85; 95% CI, 2.58–5.75), and use of diuretics (OR, 1.68; 95% CI, 1.09–2.61) (Table 4).

**TABLE 4:** Univariate and Multivariate analysis of factors associated with severe hypomagnesemia in 187 Cases with proton pump inhibitor (PPI)-related severe hypomagnesemia compared with 567 controls on PPI use without document hypomagnesemia.

	Control N = 567	Cases N = 189	Odds ratio (95% CI) univariate analysis	*p*	Odds ratio (95% CI) multivariate analysis	*p*
Age	65.9 ± 14.5	71.8 ± 11.0	1.04 (1.02–1.05)	<0.001		
Female	221 (39.0%)	103 (54.5%)	1.85 (1.35–2.61)	<0.001	1.73 (1.17–2.57)	0.006
Ethnicity (Non-Chinese vs. Chinese)	385 (67.9%)	113 (59.8%)	0.70 (0.50–0.99)	0.042		
BMI	24.9 ± 3.9	23.8 ± 4.3	0.95 (0.92–0.99)	0.001	0.90 (0.86–0.94)	<0.001
High Dose (vs. Standard PPI)	327 (57.7%)	141 (74.6%)	2.10 (1.46–3.04)	<0.001	1.96 (1.29–2.98)	0.002
Omeprazole (vs. other PPI)	546 (96.3%)	185 (97.9%)	1.79 (0.61–5.27)	0.29		
Duration of PPI (months)	43.0 (0–124)	57 (0–114)	1.0 (0.99–1.00)	0.006		
Renal impairment (eGFR<60)	128 (22.6%)	122 (64.6%)	4.21 (2.88–6.16)	<0.001	3.85 (2.58–5.75)	<0.001
Use of diuretics	98 (17.3%)	75 (39.7%)	3.12 (2.17–4.49)	<0.001	1.68 (1.09–2.61)	0.020
Diabetes Mellitus	199 (35.1%)	142 (75.1%)	5.56 (3.83–8.06)	<0.001	4.62 (3.05–7.00)	<0.001

Variables included in the multivariate analysis were age, gender, ethnicity, BMI, PPI, dosage, duration of PPI, renal impairment, use of diuretics and presence of diabetes.

BMI, body mass index; PPI, proton pump inhibitor; eGFR, estimated glomerular filtration rate.

## Discussion

In our study, we found that long-term PPI use was implicated in half of the patients with severe hypomagnesemia. Despite this, PPI therapy was stopped in less than a quarter of the patients. In addition to hypomagnesemia, PPI are also associated with other adverse effects, such as osteoporosis and renal impairment (Al-Aly et al., 2020), leading to increased calls for deprescription of PPI (Clarke et al., 2022). However, a third of our patients continued their PPI therapy despite no compelling indication for long-term PPI. When compared to controls on long-term PPI without hypomagnesemia, we also identified female gender, renal impairment, use of diuretics and dose of PPI as risk factors for PPI-related hypomagnesemia.

In our study, 66.7% (240 of 360) of patients with severe hypomagnesemia were taking PPI prior to admission, with 52.5% (189 of 360) being attributed to PPI use (128 possible, 59 probable, two definite). While two previous studies similarly found a high proportion of patients with hypomagnesemia using PPI, 54.5% (Koulouridis et al., 2013) and 41.4% (Zipursky et al., 2014), they did not assess for causality in these patients. Many of our patients had other risk factors for developing hypomagnesemia, such as use of diuretics and renal impairment. However, in 25.7% of our cases, there was no other known trigger for hypomagnesemia, such as renal or gastrointestinal losses, which strengthens the likelihood of causality. To assess for likelihood of adverse drug reactions, various algorithms are available. The Naranjo algorithm compares favourably with other algorithms like the World Health Organization-Uppsala Monitoring Center (WHO-UMC) system, or Liverpool algorithm (Gallagher et al., 2011). Unlike the WHO-UMC system, which provides a global score, the Naranjo algorithm allows a more objective measure of drug-related adverse events by assigning fixed scores, thereby reducing inter-rater variability. Because we only included patients with severe hypomagnesemia, this may explain the high proportion of PPI-related hypomagnesemia. We were conservative in our analysis, such as excluding patients if hypomagnesemia occurred prior to the first use of PPI, and it is possible that the prevalence of PPI-related hypomagnesemia could be higher. We only identified two patients with ‘definite’ PPI-related hypomagnesemia. This is because algorithms, such as Naranjo algorithm and WHO-UMC system, put a high weightage for recurrence of an adverse event after drug re-challenge. However, re-challenge with PPI was uncommon if drug causality was clear, especially with the concern of causing arrhythmias or seizures with severe hypomagnesemia.

On multi-variate analysis, we identified several factors associated with increased risk of hypomagnesemia, including use of diuretics, renal impairment, diabetes mellitus, higher dose of PPI, female gender, and lower BMI. Diuretics and renal impairment both cause hypomagnesemia *via* renal losses. Conversely, PPI use has also been associated with acute kidney injury, acute interstitial nephritis or chronic kidney disease (Al-Aly et al., 2020). Diabetes mellitus may cause hypomagnesemia, *via* poor dietary intake, increased renal losses, impaired insulin secretion and insulin resistance (Gommers et al., 2016). Meta-analyses have shown that use of PPI is associated with increased risk of hypomagnesemia, with higher dose of PPI a risk factor (Park et al., 2014; Cheungpasitporn et al., 2015; Srinutta et al., 2019). We similarly found an increased risk with higher doses of PPI, supporting the causality link between PPI use and hypomagnesemia. In view of this, clinicians may consider using the lowest required dose in patients with a strong indication for continued PPI use (Freedberg et al., 2017). We did not find duration of PPI to be associated with greater risk, but this may be due to our choice to include only patients with at least 3 month use of PPI (Kieboom et al., 2015). In one previous study, omeprazole and pantoprazole were found to have higher risk for PPI-related hypomagnesemia compared to other PPI (Luk et al., 2013). Since the cost of omeprazole is subsidized locally, a large majority of our patients were using omeprazole. We did not find any difference in risk between omeprazole and other non-omeprazole PPI. Interestingly, we found that females were more likely to develop hypomagnesemia, with 54.5% of our cases being females. Previous studies similarly found a higher proportion of females amongst cases with PPI-related hypomagnesemia, of 53.1% (Janett et al., 2015) and 52.8% (Luk et al., 2013). However, the latter study analysed adverse events reported to the FDA and found female gender to be associated with a lower risk of hypomagnesemia. A possible explanation is that they compared cases reporting of PPI-related hypomagnesemia, with controls reporting of any PPI-related adverse events, and females may be more likely to develop other PPI-related adverse events such as fractures (Thaler et al., 2016). We also found an association of PPI use with fractures (Nehra et al., 2018) but not dementia (Supplementary Table S1), and consistent with recent studies (Khan et al., 2020).

We also found that only a minority of patients prescribed with PPI develop severe hypomagnesemia. In our single referral center serving a population of ∼700,000 individuals, we had 158,523 patients prescribed with PPI over 4 years. During that same period, 189 patients had possible PPI-related severe hypomagnesemia, providing a crude estimate of 0.12%. This is slightly below a previous estimate of ∼1% (Luk et al., 2013), and likely because we included only severe hypomagnesemia (<0.4 mmol/L). This low incidence may explain why studies comparing magnesium levels in PPI users *versus* non-PPI users have either found no difference (Chowdhry et al., 2018), or marginally lower magnesium levels of 0.022 mmol/L (Kieboom et al., 2015). It is possible that patients who develop hypomagnesemia have a genetic predisposition. Hess and others found two common single nucleotide polymorphism, rs3750425 and rs2274924, in patients with PPI-associated hypomagnesemia (Hess et al., 2017). Despite PPI being contributory to severe hypomagnesemia in almost half of our patients, it was alarming that PPI were only stopped in a minority of patients. It is possible that clinicians may have attributed hypomagnesemia to other possible etiologies for hypomagnesemia that were coexistent, such as renal impairment or diuretics. In medical practice, PPI are very commonly prescribed; Severe hypomagnesemia is known to cause life-threatening arrythmia and seizures and it will be wise to prevent such episodes (Chrysant, 2019). In ∼30% of our patients, there was no indication for long-term PPI, such as DAPT or previous gastrointestinal bleeding (Freedberg et al., 2017). In addition, many patients with indications for long-term PPI were also taking higher than standard dose of PPI. However, in 42 of 43 patients where PPI was stopped, patients were prescribed histamine two receptor antagonist instead, suggesting that they had ongoing symptoms requiring treatment. In light of this, we suggest that if hypomagnesemia occurs during the course of PPI therapy, it will be prudent for clinicians to review its indication from time to time, or considering switching to histamine two receptor antagonist (Al-Aly et al., 2020).

Urinary magnesium levels in our patients were generally low. This supports the current hypothesis that PPI impair magnesium absorption, in particular active uptake of magnesium (Hess et al., 2012; Janett et al., 2015). Since magnesium absorption can also occur *via* a passive paracellular pathway, this explains why in many of our patients, magnesium supplementation alone corrected hypomagnesemia rapidly, despite continuation of PPI. However, only 1% of total body magnesium is present in blood, with the remaining 99% in bones, muscles and soft tissue (Elin, 2010). Hence, even after early normalisation of magnesium levels, these patients may have a significant total body magnesium deficit. To illustrate this, several of our patients (Supplementary Figure S2) had recurrences of hypomagnesemia, and complete normalisation occurred months later. Hypomagnesemia also improved when patients switched to a histamine two receptor antagonist, consistent with other reports that hypomagnesemia is due to a class effect of PPI (Hess et al., 2012; Janett et al., 2015).

The present study had several limitations. First, it was a retrospective study, and management of hypomagnesemia was not standardised. A prospective study with a protocol for stopping or reducing PPI dose may guide the optimal management of patients with PPI-related hypomagnesemia. Second, the temporal relationship (cause-effect) of PPI with development of hypomagnesemia could not be clearly demonstrated in all patients. However, the Naranjo algorithm includes an item which scores patients with documented normomagnesemia prior to PPI use, and development of hypomagnesemia only after PPI use. 53% of patients fulfilled this criterion which supports the temporal relationship of PPI-related hypomagnesemia. Third, we elected to focus only on severe hypomagnesemia which was more clinically relevant. Hence, we were not able to evaluate the impact of milder degrees of hypomagnesemia. Finally, all cases with severe hypomagnesemia were admitted for treatment, whereas controls were selected from all patients prescribed PPI in the same time period, potentially leading to differences in the two groups. However, the risk factors we identified (renal impairment and use of diuretics) are consistent with other studies and we did not find differences in unrelated conditions such as gastritis or peptic ulcer.

## Conclusion

We have found that use of long-term PPI played a contributory role in almost half of all patients with severe hypomagnesemia. Although magnesium levels transiently returned to normal after supplementation, recurrence were frequent in patients continued on PPI. Clinicians need to be aware of this uncommon but serious side-effect of PPI, and constantly review the indication to continue long-term PPI to prevent potential hypomagnesemia episodes which can lead to life-threatening arrythmias or seizures.

## Data Availability

The raw data supporting the conclusion of this article will be made available by the authors, without undue reservation.
