# Supplementary material for: Proton-pump inhibitor use amongst patients with severe hypomagnesemia
Source: Front Pharmacol. 2023 Jan 30;14:1092476. doi: 10.3389/fphar.2023.1092476 (PMC9922884; doi:10.3389/fphar.2023.1092476)
Supplement: Supplementary file 1 [file DataSheet1.PDF]

Supplementary text

Methods

For items 1 and 10, all patients received 1 point, as there have been previous conclusive reports in the literature (item 1), and all patients had an objective measurement (item 10) with serum magnesium levels to confirm the adverse drug reaction. There were no points given for item 6 and 7 in any patients, as no patients received a placebo (item 6) and no measurements were made for toxic levels of PPI in bodily fluids (item 7). For item 2, patients had to have documented normal magnesium levels prior to initiation of PPI, and development of hypomagnesemia after PPI initiation, to be scored yes (2 points). For item 3, patients were required to have documented normal magnesium levels after PPI discontinuation to be scored yes (1 point). For item 4, only patients re-challenged with PPI could be evaluated for response, with recurrence (2 points), or no recurrence (-1 point). For item 5, all patients were assessed for other possible etiologies for hypomagnesemia. This included any evidence of gastrointestinal losses such vomiting or diarrhea prior to hypomagnesemia, chronic renal impairment (defined as calculated glomerular filtration rate below 60ml/min per 1.73 m^2, poorly controlled diabetes (defined as HbA1C >9%), pancreatitis, alcoholism, hypercalcemia, use of diuretics or other medications such as calcineurin inhibitors, cisplatin, and gentamicin. For item 8, occurrence or resolution of hypomagnesemia after changes in dose of PPI were scored accordingly (yes, 1 point; no, 0 points). For item 9, patients with more than one episode of hypomagnesemia during PPI therapy were scored yes (1 point) (*supplementary text*).

Other electrolytes and urine magnesium levels were noted if available. Complications that could be attributed to hypomagnesemia, including hypocalcemia, hypokalemia, arrhythmias and seizures were recorded. Any episodes of milder hypomagnesemia prior to, or after, this episode, were noted.

Supplementary Table S1. Clinical characteristics of Cases (Patients PPI-induced severe hypomagnesemia) compared with controls (Patients on chronic PPI use without document hypomagnesemia

	Control<br>N=567	Cases<br>N = 189	*P*
Age	66.0 (47 - 85)	73.0 (58 - 88)	0.006
Female	221 (39.0%)	103 (54.5%)	<0.001
Race<br>- Chinese<br>- Malay<br>- Indian<br>- Others	 385 (67.9%)<br>114 (20.1%)<br>44 (7.8%)<br>24 (4.2%)	 113 (59.8%)<br>51 (27.0%)<br>14 (7.4%)<br>11 (5.8%)	0.152
BMI	24.8 $\pm$ 5.3 (n = 406)	23.4 $\pm$ 5.4 (n = 142)	0.529
Type PPI<br>-Omeprazole<br>-Non-omeprazole	 544 (96.3%)<br>11 (3.7%)	 185 (97.9%)<br>4 (2.1%)	0.493
Standard Dose PPI	234 (41.7%)	48 (25.4%)	<0.001
Duration of PPI, months	43.0 (3 – 124)	57 (3 - 114)	0.006
Na	142.0 $\pm$ 67.8 (n = 337)	142.5 $\pm$ 94.6 (n = 174)	<0.001
K	4.2 $\pm$ 0.5 (n = 356)	4.3 $\pm$ 5.7 (n = 174)	<0.001
Ca	2.3 $\pm$ 0.2 (n = 179)	2.0 $\pm$ 0.4 (n = 163)	<0.001
CKD	76 (13.5%)	60 (31.7%)	<0.001
Cirrhosis	2 (0.4%)	6 (3.2%)	0.001
DM	199 (35.2%)	142 (75.1%)	<0.001
AF	70 (12.4%)	25 (13.2%)	0.764
COPD	21 (3.7%)	10 (5.3%)	0.345
HT	373 (66.0%)	165 (87.3%)	<0.001
HL	337 (59.6%)	137 (72.5%)	0.002
IHD	260 (46.0%)	125 (66.1%)	<0.001
PVD	5 (0.9%)	16 (8.5%)	<0.001
CVA	60 (10.6%)	54 (28.6%)	<0.001
CCF	37 (6.5%)	36 (19.0%)	<0.001
Gastritis	107 (18.9%)	40 (21.2%)	0.504
PUD	22 (3.9%)	18 (9.5%)	0.003
Dementia	38 (6.7%)	19 (10.1%)	0.134
Fractures	32 (5.7%)	21 (11.1%)	0.011
Pancreatitis	7 (1.2%)	1 (0.5%)	0.410
Alcoholism	6 (1.1%)	4 (2.1%)	0.273
Charlson’s CI only	3 (0 - 6)	6 (3 - 9)	<0.001
Aspirin	325 (57.7%)	107 (56.6%)	0.789
Diuretics	98 (17.4%)	75 (39.7%)	<0.001

Data presented as mean (SD), median (interquartile range), or number (percentage).

Supplementary Figure S1a, Levels of Mg and K in cases

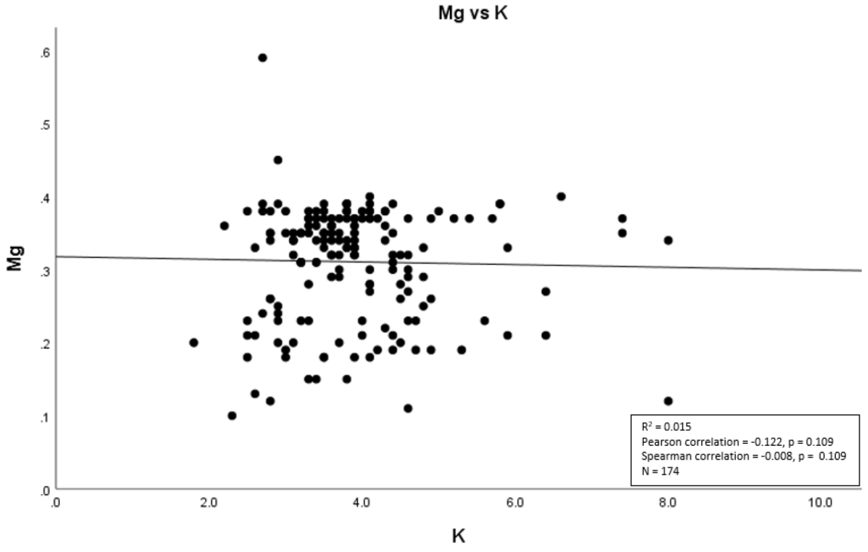

Supplementary Figure S1b, Levels of Mg and Ca in cases

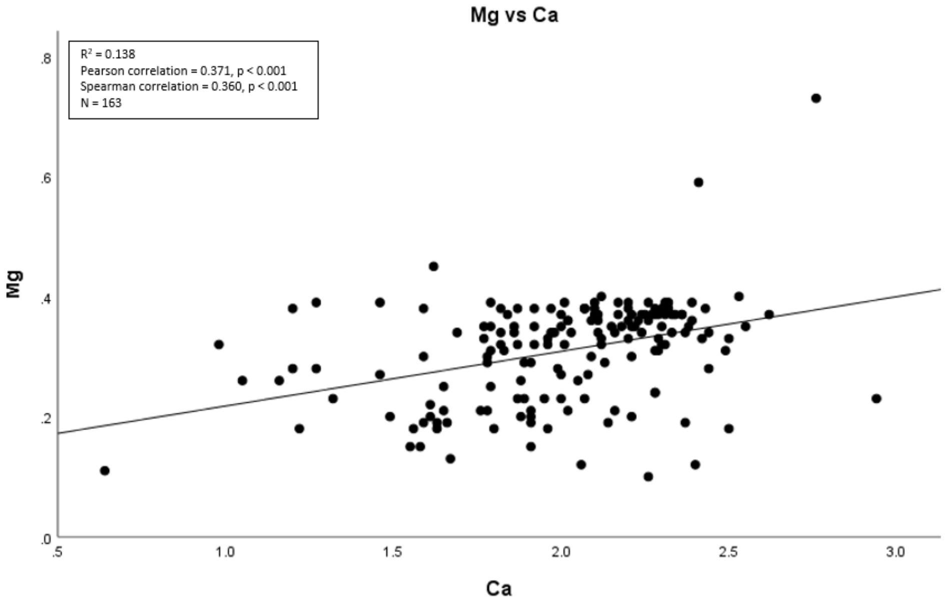

Supplementary Figure S2. Three Case illustrations and Naranjo Scores

Case 1

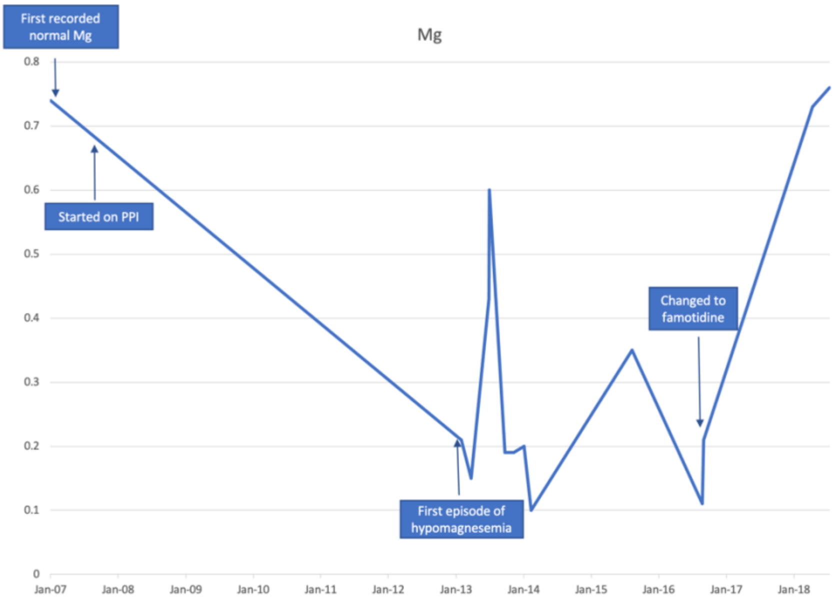

**Naranjo questionnaire**	**Score**	
1. Are there previous conclusive reports on this reaction?	Yes	+1
2. Did the adverse events appear after the suspected drug was given?	Yes	+2
3. Did the adverse reaction improve when the drug was discontinued or a specific antagonist was given?	Do not know	0
4. Did the adverse reaction appear when the drug was readministered?	Do not know	0
5. Are there alternative causes that could have caused the reaction?	Yes	-1
8. Was the reaction more severe when the dose was increased, or less severe when the dose was decreased?	Do not know	0
9. Did the patient have a similar reaction to the same or similar drugs in any previous exposure?	Yes	+1
10. Was the adverse event confirmed by any objective evidence?	Yes	+1
**Total**	4, Possible	

Case 2

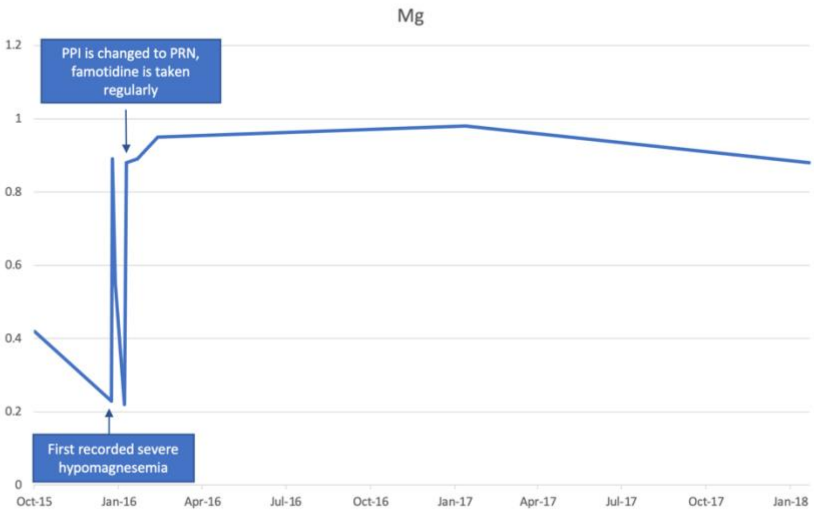

**Naranjo questionnaire**	**Score**	
1. Are there previous conclusive reports on this reaction?	Yes	+1
2. Did the adverse events appear after the suspected drug was given?	Do not know	0
3. Did the adverse reaction improve when the drug was discontinued or a specific antagonist was given?	Yes	+1
4. Did the adverse reaction appear when the drug was readministered?	Do not know	0
5. Are there alternative causes that could have caused the reaction?	Yes	+2
8. Was the reaction more severe when the dose was increased, or less severe when the dose was decreased?	Do not know	0
9. Did the patient have a similar reaction to the same or similar drugs in any previous exposure?	Yes	+1
10. Was the adverse event confirmed by any objective evidence?	Yes	+1
**Total**	6, Probable	

Case 3

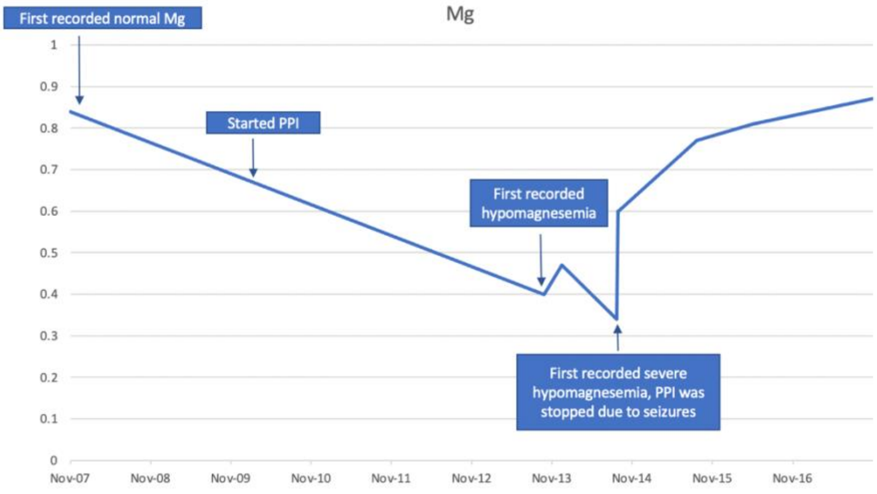

Naranjo questionnaire	Score	
1. Are there previous conclusive reports on this reaction?	Yes	+1
2. Did the adverse events appear after the suspected drug was given?	Yes	+2
3. Did the adverse reaction improve when the drug was discontinued or a specific antagonist was given?	Yes	+1
4. Did the adverse reaction appear when the drug was readministered?	Do not know	0
5. Are there alternative causes that could have caused the reaction?	Yes	+2
8. Was the reaction more severe when the dose was increased, or less severe when the dose was decreased?	Do not know	0
9. Did the patient have a similar reaction to the same or similar drugs in any previous exposure?	Yes	+1
10. Was the adverse event confirmed by any objective evidence?	Yes	+1
**Total**	8, Probable	
